# Downregulation of AKT/mTOR signaling pathway for *Salmonella*-mediated autophagy in human anaplastic thyroid cancer

**DOI:** 10.7150/jca.75163

**Published:** 2022-09-06

**Authors:** Li-Hsien Wu, Christian R. Pangilinan, Che-Hsin Lee

**Affiliations:** 1 Department of Biological Sciences, National Sun Yat-sen University, Kaohsiung 80424, Taiwan.; 2 School of Medicine, College of Medicine, National Sun Yat-sen University, Kaohsiung 80424, Taiwan.; 3 Department of Medical Research, China Medical University Hospital, China Medical University, Taichung 404, Taiwan.; 4 International PhD Program for Science, National Sun Yat-sen University, Kaohsiung 80424, Taiwan.; 5 Aerosol Science Research Center, National Sun Yat-sen University, Kaohsiung 80424, Taiwan.

**Keywords:** anaplastic thyroid cancer, *Salmonella*, Autophagy, AKT/mTOR

## Abstract

Thyroid cancer has been known as the most common endocrine malignancy. Although majority of thyroid cancer types respond well to conventional treatment including surgery and radioactive iodine therapy, about 10% of those with differentiated thyroid cancer will present distant metastasis and will have persistent or recurrent disease. Even more serious is a rare type of thyroid cancer called anaplastic thyroid cancer (ATC), which accounts for about 1%, has been demonstrated as the most lethal and aggressive form of human malignancy. Unfortunately, these tumors are also frequently resistant to traditional therapy. Previous study have shown that *Salmonella* inhibits tumor growth, in part, by inducing autophagy - a cellular process that is important in the innate and adaptive immunity in response to viral or bacterial infection. In our study, we intended to investigate whether *Salmonella* can inhibit tumor growth by inducing autophagy, specifically in thyroid cancer and elucidate the possible molecular mechanism. In order to determine the signaling pathway involved in tumor cell autophagy, we used *Salmonella* to treat ATC cells line ASH-3 and KMH-2 *in vitro*. The autophagic markers, particularly autophagy-related gene 6 (Beclin-1), microtubule-associated protein 1A/1B-light chain 3 (LC3) and p62, were observed to be differentially expressed after infection with *Salmonella* indicating an activated autophagy in ATC cells. In addition, the protein expression levels of phospho-protein kinase B (P-AKT), phospho-mammalian targets of rapamycin (P-mTOR), phospho-p70 ribosomal s6 kinase (P-p70S6K) in tumor cells were decreased after *Salmonella* infection. *In vivo*, we also found that substantial cell numbers of *Salmonella* targeted tumor tissue, and regulated anti-tumor mechanisms. Our findings showed that *Salmonella* activated autophagic signaling pathway and inhibited ATC tumor growth via downregulation of AKT/mTOR pathway.

## Background

Thyroid cancer is the most common endocrine malignancy with about 550,000 annual recorded cases worldwide and its incidence keep on increasing. About 90% of thyroid cancers are differentiated thyroid cancers (DTC) and has an outstanding 10-year survival rate about 95% 1. The mainstream treatment for most thyroid cancer patients is surgery and often involves radioactive iodine (RAI) as an adjuvant treatment 2. Although most patients have a good prognosis and a reasonably high survival rate, about 10% of patients with DTC may develop distant metastases and about 6-20% will have disease recurrence at distant sites 3. Unfortunately, this recurrent state of the cancer is poorly differentiated which have higher rate of resistance to iodine and insensitive to traditional cytotoxic chemotherapy agents 4. In addition, anaplastic thyroid cancer (ATC) is a rare type of thyroid tumor which accounts for about 1% of thyroid cancer cases 5. ATC has been demonstrated to be the most lethal and aggressive form among human malignancies. Aside from being the most aggressive thyroid cancer, ATC also has the characteristics of rapid growth. This means, the mass in the neck of patients with ATC may rapidly expand and metastasize, resulting in airway and esophageal obstruction 6, 7. The most common metastatic sites are the lungs, followed by the bones and the brain. Despite the use of surgical resection, chemotherapy, and radiation, the prognosis of ATC patients remains poor, with an average survival rate of only 6 months 8. Hence, the current research on the disease, mechanism, and effective treatment methods for ATC still needs to be done 9, 10. Therefore, targeting drugs or immunotherapy for some signaling pathways might be more prospective thyroid cancer treatment strategies.

In this study, we chose 2 ATC cell lines to determine the effect of *Salmonella* treatment against ATC and explore the cellular mechanism involved in *Salmonella*-mediated tumor growth inhibition. Because of the preferential colonization and proliferative capacity of the facultatively anaerobic *Salmonella*, as well as its potential to prevent metastasis, it is considered as the most favorable agent in bacterial-mediated cancer therapy 11, 12. *Salmonella* can reduce tumor metastasis by impacting the tumor microenvironment, including in melanoma, breast cancer and lung carcinoma 13-16. Our previous studies have shown that *Salmonella* can downregulate Matrix Metalloproteinases-9, C-X-C chemokine receptor type 4, heparinase and SNAI1 expression to reduce tumor metastasis. Other studies have indicated that *Salmonella* can enhance its therapeutic potential by increasing tumor targeting and improving safety 17, 18. Because of these advantages, many studies studied about the combinations of* Salmonella* and other approaches for cancer therapy, including radiotherapy, chemotherapy, and immune checkpoint inhibitors, to enhance the therapeutic efficacy 18-20. These studies have successfully shown the use of *Salmonella* regulate the tumor microenvironment to suppress tumor, which means the potential to change the tumor microenvironment from immunosuppressive to immunogenic environment. This is mainly achieved by changing the cellular and soluble components of the immune system and affecting the phenotypic and functional characteristics of immune cells. *Salmonella* can enhance the immune response to tumor cells by increasing the tumor-infiltration of different innate and adaptive immune cells such as CD4^+^ helper T cells, CD8^+^ cytotoxic T cells, B cells, macrophages, neutrophils and natural killer (NK) cells 21-23. Previous research had shown that the amount of *Salmonella typhimurium* in tumor was 1000 times more than normal tissues or organs, including spleen and liver, showing that *Salmonella* overcomes the limitations of insufficient specificity and tissue penetration in conventional therapies 12, 24. Former studies indicated that *Salmonella* can inhibit tumor growth by inducing autophagic signaling pathway in tumor cells 25. Autophagy is a self-degradative mechanism that disassembles the long-lived proteins and unnecessary organelles in the cytosol. When cells are under stressful conditions such as hypoxia, nutrient deprivation or infection, autophagy is induced to regulate the balance of cell survival by a multitude of factors including nutritional status, hormones and intracellular signaling pathways 26-28. In malignancies, autophagic activity is generally low, and *Salmonella* activates cell death pathways through nutrient competition and stimulation of tumor-specific immune responses. Additionally, phosphoinositide 3 kinase (PI3K)/protein kinase B (AKT)/ mammalian targets of rapamycin (mTOR) is a conventional autophagy pathway 29. In our previous study found that *Salmonella* regulated the autophagic signaling pathway through downregulation of AKT/mTOR pathway and control the melanoma growth 25.To date, ATC is still poorly understood. Targeting specific genetic alterations and signaling pathways in patients with ATC remains an attractive cancer treatment strategy. Since it is known that *Salmonella* treatment may alter protein expressions of infected cells, we used this bacterial agent in this study to observe its effects on tumors *in vivo* and *in vitro,* particularly to dissect the mechanism whether autophagy plays an essential role in the context of ATC growth inhibition.

## Materials and methods

### Cell lines, bacteria, and mice

Human anaplastic thyroid carcinoma cell lines ASH-3 and KMH-2 were obtained from Dr. Ying-Ray Lee (Kaohsiung Medical University, Taiwan). ASH-3 were cultured in DMEM+RPMI 1640 (1:1) media (GIBCO Inc.) supplemented with 10% fetal bovine serum (FBS, Sigma-Aldrich Co.) and 1% penicillin/ streptomycin (Sigma-Aldrich Co.) in cell incubator with 5% CO_2_ (SANYO Co., Japan). The *Salmonella enterica* serovar* choleraesuis* (*S*. Choleraesuis; S.C.) (ATCC 15480) vaccine strain was received from the Bioresources Collection and Research Center (Hsinchu, Taiwan). This crude variant of S.C., called Vaccine 51, was attained by spreading an 18-hour broth culture of malign strain 188 of S.C. serotype Dublin over the face of a dried nutrient agar plate, adding a drop of a suspension of *Salmonella* anti-o phage No. 1 and opting for a phage-resistant colony after incubation at 37 °C for 24h 30,31. Four-to-five-week-old female BALB/c nude (BALB/cAnN.Cg-*Foxn1*^nu^/CrlNarl) mice were purchased from the National Laboratory Animal Center of Taiwan. The animals were maintained in a specialized pathogen-free animal care facility and fed by standard sterile chow diet. Mice followed by isothermal conditions with regular photoperiods. The experimental protocol adhered to the rules of the Animal Protection Act of Taiwan and was approved by the Laboratory Animal Care and Use Committee of the National Sun Yat-Sen University (permit number: 10829).

### Plasmid Transfection

To examine autolysosome and autophagosome constitution in cells, 5ug of GFP-LC3 plasmid was transfected using Lipofectamine 2000 (Invitrogen, Carlsbad, CA, USA) 32. In addition, to confirm the role of the AKT pathway, the constitutively active AKT plasmid which was kindly provided by Dr. Chiau-Yuang Tsai (Department of Molecular Immunology, Osaka University) were transfected using Lipofectamine 2000 according to the manufacturer's instructions 33. The transfected cells were infected with *Salmonella* or PBS control for 90 min. The expression of AKT pathway was analyzed by Western blotting and the fluorescence of GFP-LC3 was visualized by fluorescence microscopy.

### Cell viability assay

Cells (2 × 10^4^ cells/well) were seeded in 96-well cell culture plates, then treated by PBS or *Salmonella* (1-100 multiplicity of infection; MOI) for 1.5 hours. Cell viability was examined using the WST1 assay kit (Roche, West Sussex, UK) and the optical density was measured using the SPECTROstar Nano Microplate Reader (BMG LABTECH, Germany) at a specified time.

### Cell proliferation assay

The cells were seeded in 96-well culture plates (10^4^/well), then treated by PBS or *Salmonella* (1-100 multiplicity of infection; MOI) for 1.5 hours. Cell proliferation was examined with the 5-bromo-2'-deoxyuridine (BrdU) Cell Proliferation Fluorescence Imaging Kit (AAT Bioquest, US) uses BrdU which was incorporated into cellular DNA during DNA synthesis. After fixing cells, the incorporated BrdU is labelled with iFluor® 488 MTA. The images were taken with the Olympus fluorescence microscopy.

### Cell death assay

The ASH-3 and KMH-2 cells (5×10^5^ cells/well) were placed into 6-well plates and incubated at 37 °C for 24 hours and then the cells were infected with 100 MOI of *Salmonella* for 90 min. ATC cells were collected after 24 hours. Cells were processed with the AnnexinV-Fluorescein isothiocyanate (FITC) Apoptosis Detection Kit (Strong Biotech Co., Taipei, Taiwan) according to the manufacturer's instructions. The fluorescence of 10,000 cells per sample was measured and analyzed with Attune NxT Flow Cytometer (Life Technologies, Carlsbad, CA, USA).

### Western blotting

The protein sample concentration was measured by Bicinchoninic Acid Protein Assay (ThermoFisher, Waltham, MA, USA). Proteins were subjected to SDS-PAGE separation, and the separated proteins were transferred to PVDF membrane (Pall Corporation, East Hills, NY, USA). The membrane was blocked with primary antibodies p62 (Novus Biologicals, Littleton, CO, USA), LC3 (Novus Biologicals), beclin-1 (Novus Biologicals), p70S6 kinase (p70S6K) (Cell Signaling, Danvers, MA, USA), AKT (Santa Cruz Biotechnology, Inc. Santa Cruz, CA, USA), mTOR (Cell Signaling), phosphor-AKT (Santa Cruz Biotechnology, Inc.), phosphor-mTOR (Cell Signaling), phosphor-p70S6K (Cell Signaling) or monoclonal antibodies against b-actin (AC-15, Sigma Aldrich). Horseradish peroxidase-conjugated goat anti-mouse IgG or anti-rabbit IgG (Jackson, West Grove, PA, USA) was used as the secondary antibody and protein-antibody complexes were visualized by enhanced chemiluminescence system (Amersham). The signals were quantified with ImageJ software (rsbweb.nih.gov/ij).

### Animal studies

Tumor cells (1×10^7^ cells/mouse) were mixed with Matrigel at a ratio of 1:1 to protect tumor cells growth, then injected subcutaneously into the right flank of the mice. Mice were divided into two groups: group treated with *Salmonella*; and group treated with PBS. The mice were intraperitoneal injected with 2×10^6^ cfu of *Salmonella* at day 7 post-tumor inoculation. The mice were sacrificed at various time points post infection and the numbers of *Salmonella* in the tumors, livers and spleens were determined using spread plate method on LB agar plates; the data were expressed as cfu per gram of tissue. In a separate experiment, the length (L) and width (W) of palpable tumors were measured every 3 days using calipers. The volume of palpable tumors was calculated using following formula: (length of tumor) × (width of tumor)^2^ × 0.45. The body weight was monitored every 3 days and the survival of mice were monitored daily.

### Immunohistochemistry

To analyze the cell proliferation marker proliferating cell nuclear antigen (PCNA), autophagy marker LC3-II, the expression of AKT/mTOR, and infiltrates of macrophages (F4/80) and nutrophils (Ly-6G) in tumors, BALB/c nude mice were inoculated subcutaneously ASH-3 (10^7^) and intraperitoneal injected *Salmonella* (2×10^6^ cfu) or PBS on day 0. Then tumor were excised at day 7 and day 14 then washed with PBS, fix with 3.7% formaldehyde, and embedded in paraffin. Tumor tissues were then processed in 5µm sections and stained with phosphor-AKT (Arigo Biolaboratories), phosphor-mTOR (Arigo Biolaboratories), F4/80 (Arigo Biolaboratories), lymphocyte antigen 6 complex locus G6D (Ly-6G; Arigo Biolaboratories), proliferating cell nuclear antigen (PCNA; Arigo Biolaboratories), and LC3-II (Arigo Biolaboratories) were detected by immunohistochemistry.

### Statistical analysis

All quantified data are expressed as the mean ± standard deviation (SD). Statistical significance was analyzed using one-way analysis (one-way ANOVA) of variance and the difference among the means was calculated using SigmaPlot software (System Software, San Jose, CA, USA). A survival analysis was conducted by the Kaplan-Meier survival curve and log-rank test. Statistical significance was considered when the p value is < 0.05.

## Results

### *Salmonella* inhibited growth of ATC cell lines *in vitro*

**Figure 1:**
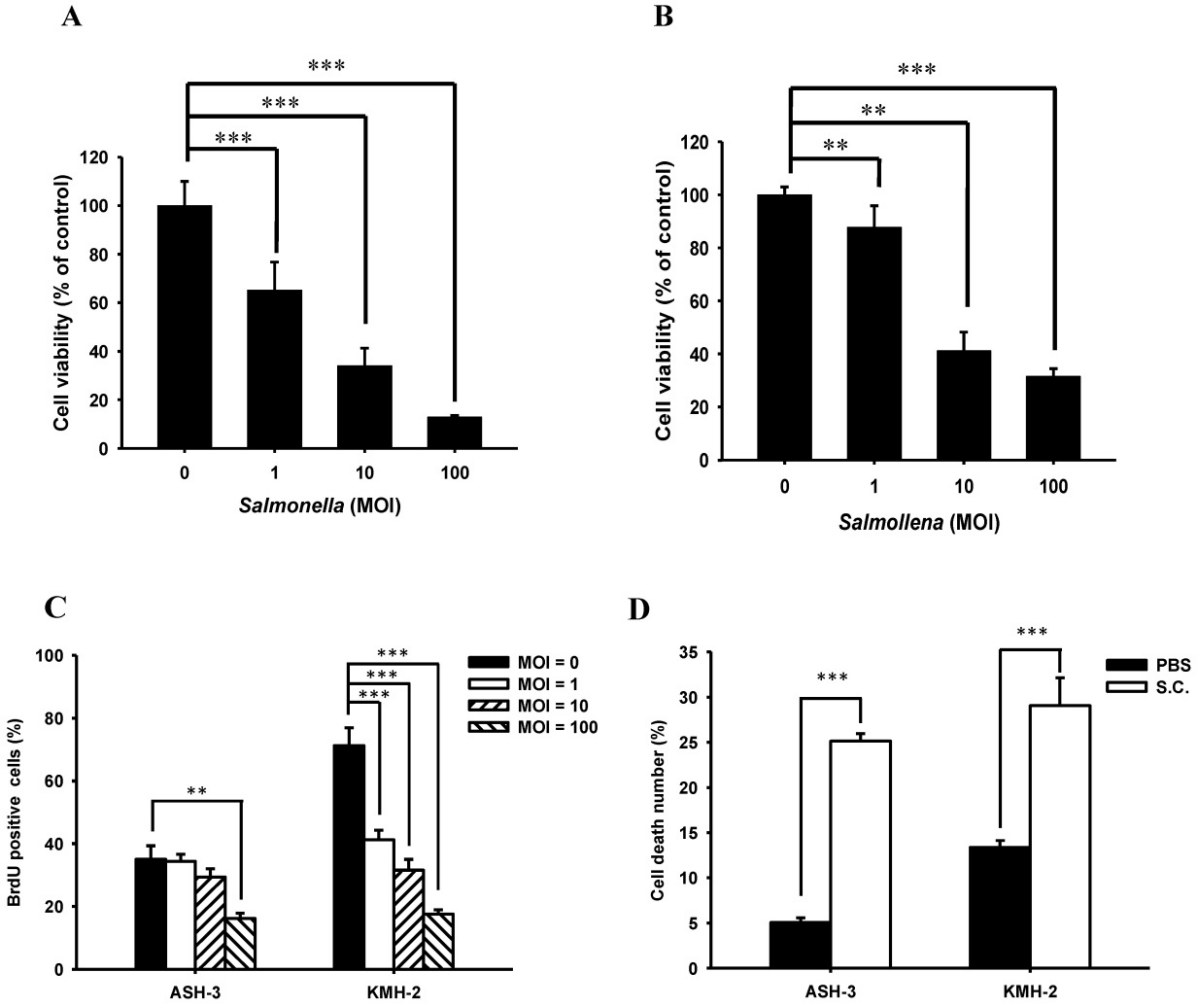
** Cell viability of ASH-3 and KMH-2 human tumor cells infected with *Salmonella*.** The ASH-3 (**A**) and KMH-2 cells (2 × 10^4^ cells/well) (**B**) were seeded into 96-well plates and incubated at 37 °C for 24 hours. After treatment with various MOI of *Salmonella* for 90 min, each well were loaded with proliferation reagent WST-1. Then, the absorbance was read and analyze to measure the cell viability (mean ± SD, n=8). (**C**) For monitoring cell proliferation, ATC cells were treated with various MOI of *Salmonella* for 90 min. Then cells were fixed and stained for BrdU immunoreactivity and nuclei were counterstained with Hoechst 33342. The cells were counted under a fluorescence microscope (mean ± SD, n=6). (**D**) After treated with *Salmonella* (100 MOI), the ASH-3 and KMH-2 cells were stained with Annexin V and the frequency of cells were examined by flow-cytometry (mean ± SD, n=6). *** *p* < 0.001; ** *p* < 0.01.

We first evaluated whether *Salmonella* would reduce proliferation of ATC *in vitro*. A concentration range of 0-100 MOI *Salmonella* were formulated to treat ASH-3 and KMH-2 cells for 90 min. The cell viability assay revealed that both ASH-3 (Figure 1A) and KMH-2 (Figure 1B) treated with *Salmonella* were significantly reduced in a dose-dependent manner compared with control. To monitor cell proliferation influence by *Salmonella* in ATC cells, we use BrdU fluorescence to investigate the range of 0-100 MOI* Salmonella* (Figure S1). Figure 1C showed that *Salmonella* inhibited cell proliferation in these cells in a dose-dependent manner by quantification of BrdU-positive cells. Furthermore, the effects of *Salmonella*-treated on cell death of ASH-3 and KMH-2 were further examined using Annexin V/PI assay (Figure S2). As shown in Figure 1D, after treatment with *Salmonella*, the number of death cells increased by approximately 20% compared to the PBS group. The results suggest that *Salmonella* inhibited ATC cells proliferation and viability.

### *Salmonella* regulated autophagy in ATC

**Figure 2:**
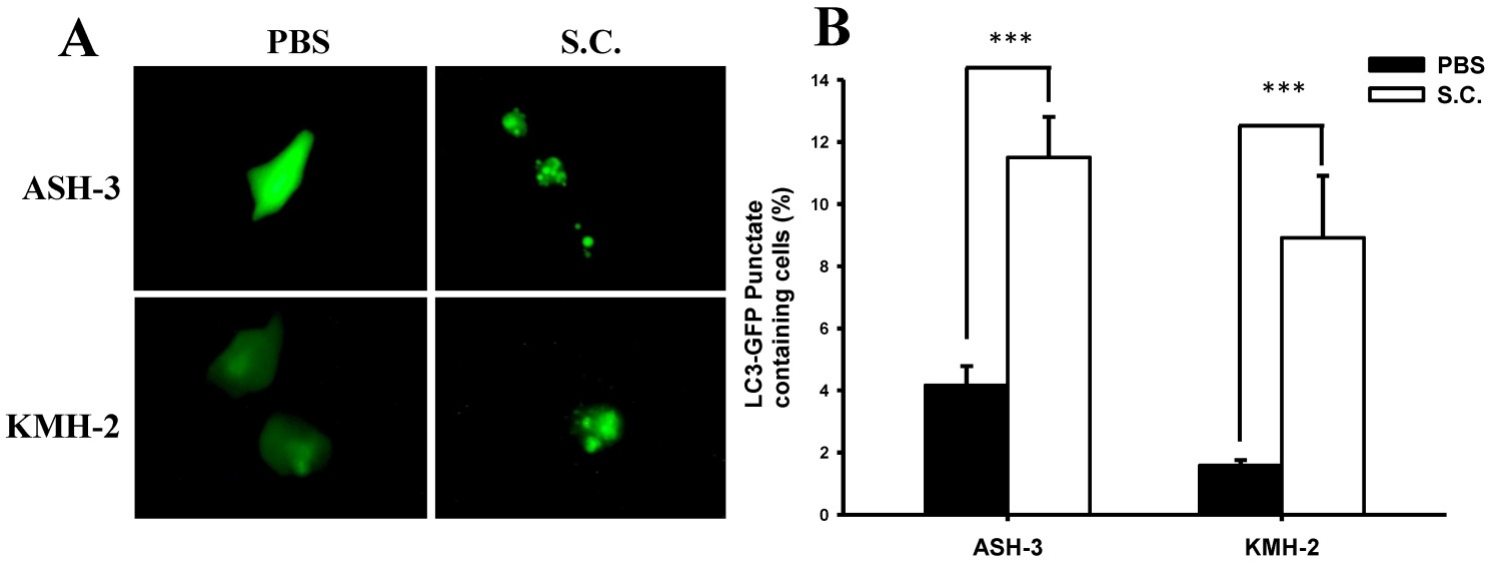
** Effect of *Salmonella* on autophagic punctate formation*.*** The ASH-3 and KMH-2 cells (10^6^ cells/well) were placed into 6-well plates and incubated at 37 °C for 24 hours. Cells were transfected with the plasmid encoding GFP-LC3 for 6 hours and then infected with *Salmonella* (10^8^ cfu) or PBS for 90 min. (**A**) The LC3 puncta were visualized by fluorescence microscopy after treatment with *Salmonella*. (**B**) Shows the quantification of autophagosome punctate percentage of cells (mean ± SD, n=11). *** *p* < 0.001.

A recent review demonstrated that *Salmonella* infection partially triggers autophagy 34. Based on our initial findings, we presume that *Salmonella* inhibited ATC cell growth, in part, by inducing autophagy. During autophagy, LC3 proteins concentrate on the surface of autophagosomes and turn into punctate distribution. Punctate fluorescence has been known as a good indicator of autophagy. To examine the induction of autophagic flux by *Salmonella*, the GFP-fused LC3 (GFP-LC3) plasmids were transfected into ASH-3 or KMH-2 cells and then the autophagosome and autolysosome puncta were located using fluorescent microscopy 32. Figure 2A shows that *Salmonella* treatment resulted in an enhanced autophagosome formation and simultaneously increased autophagic flux in ASH-3 and KMH-2 cells, whereas the PBS group showed diffuse cytoplasmic distribution state. The quantification results of the percentage of autophagosome punctuated dots in both ASH-3 and KMH-2 were significantly enhanced about 7% in *Salmonella*-treated cells compared with the PBS group (Figure 2B). The results indicated that *Salmonella* acts as an autophagy inducer in ASH-3 and KMH-2 cells which motivated us to further explore the detailed mechanism of *Salmonella* autophagy in ATC.

### Regulation of signaling pathways with *Salmonella* in ATC

**Figure 3:**
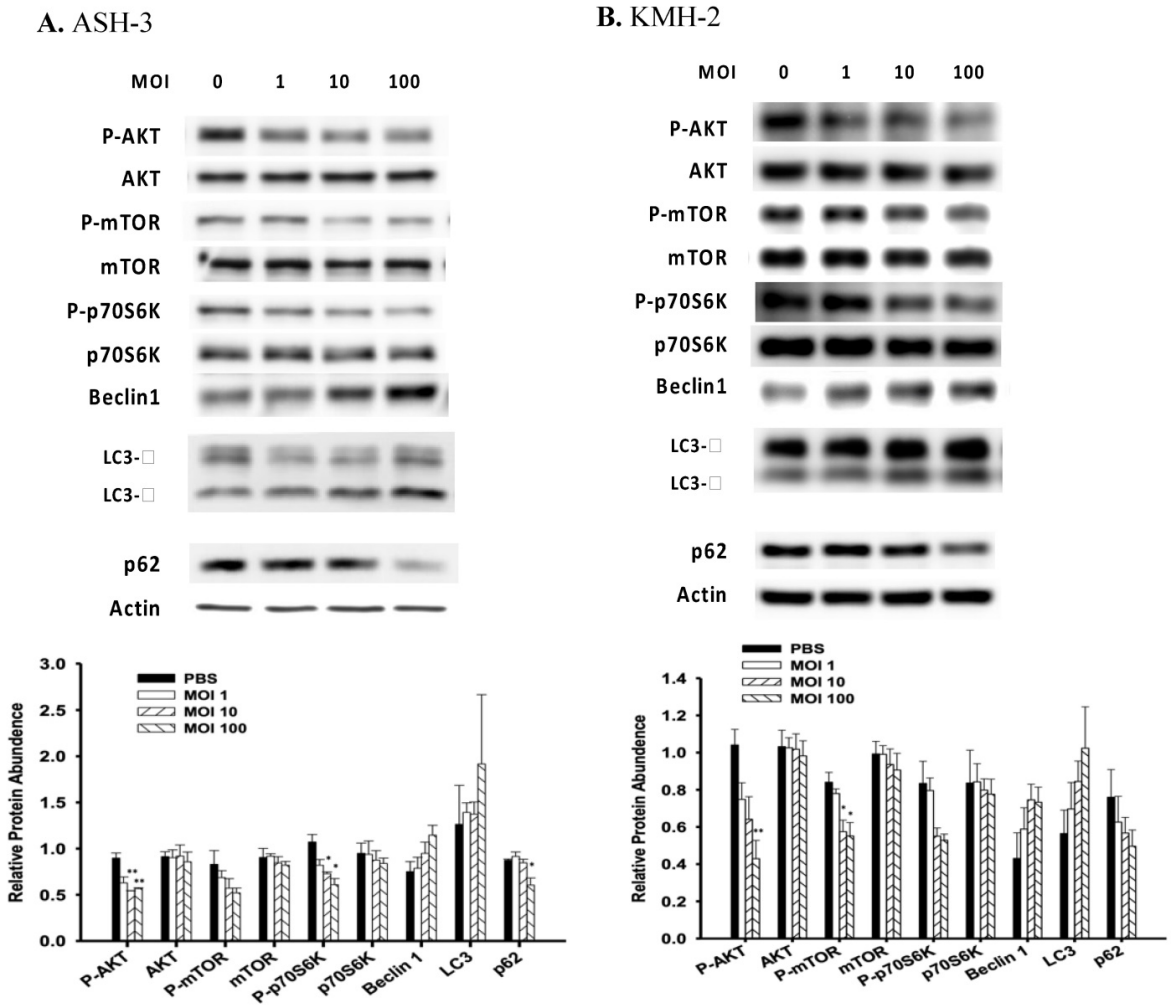
***Salmonella* induced autophagic signaling pathway.** The ASH-3 (**A**) and KMH-2 (**B**) cells (5 × 10^5^ cells/well) were placed into 6-well plates and incubated at 37 °C for 24 hours and then the cells were infected with various MOI of *Salmonella* for 90 min. The expression level of AKT/mTOR/p70S6K proteins and autophagic marker in cells were analyzed by Western blotting. β-actin was used as an internal loading control. Quantification histograms are presented beneath each Western blotting plot. Data are expressed as the mean ± SD of hexaplicate determinations. Each experiment was repeated three times with similar results.

Induction of autophagy is associated with many signaling pathways, and P-AKT is one of the pathways that negatively regulate autophagy 35. Previously, our group showed that *Salmonella* alters various signaling pathways such as mitogen-activated protein kinase (MAPK) signaling and AKT/mTOR signaling in many cancer types 36,16. To investigate the mechanisms underlying *Salmonella*-induced autophagy, the expression of AKT/mTOR/p70S6K signaling pathway were evaluated in ASH-3 and KMH-2 following *Salmonella* treatment. The results showed that the expression of AKT/mTOR/p70S6K phosphorylation was decreased in a dose-related manner in ASH-3 (Figure 3A) and KMH-2 (Figure 3B) following treatment with *Salmonella* which indicate a significant suppression of the AKT/mTOR/p70S6K phosphorylation activity by *Salmonella* in both cells. LC3-II is a marker protein of autophagy. When autophagy is activated, LC3-I is recruited to autophagosomes and then converted into LC3-II 37. The protein p62 is one of the selective substrates of autophagy, localized to autophagosomes and continuously degraded through the autophagy-lysosome pathway, thereby decreasing as autophagy activity increases 38. Beclin-1 plays a central role in autophagy, which increases during cellular stress and disappears during the cell cycle 39. Results showed that ASH-3 (Figure 3A) and KMH-2 (Figure 3B) treated with *Salmonella* increased the conversion of LC3-I to LC3-II, enhanced the expression of beclin-1 and decreased the expression of p62, suggesting a *Salmonella*-mediated induction of autophagy in both cells. These results can also be clearly observed in the quantification histogram below. Summarizing these results, we can observe that *Salmonella* activates autophagy through the AKT/mTOR/p70S6K signaling pathway in ATC.

### AKT signaling pathway contributed to *Salmonella*-induced autophagy in ATC

**Figure 4:**
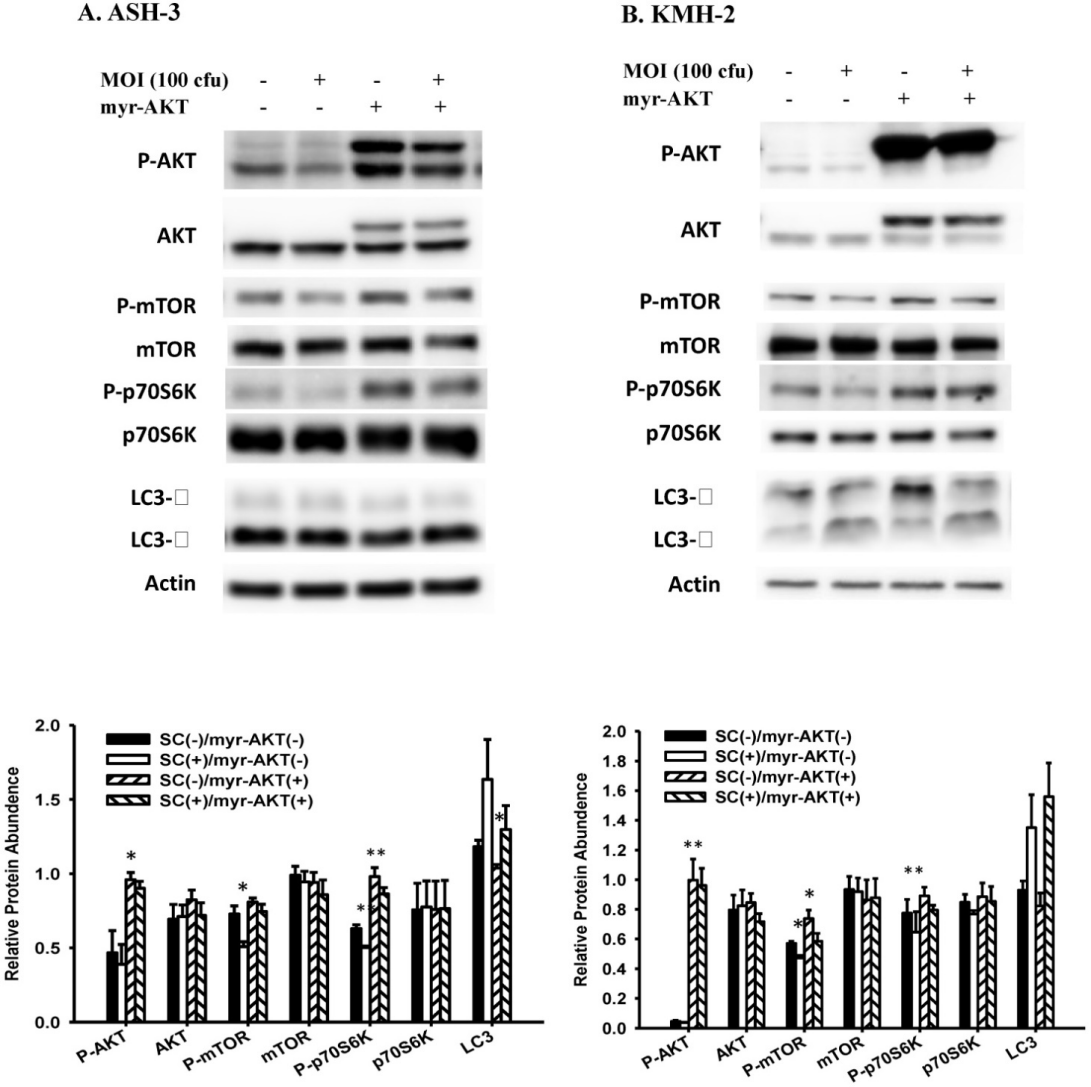
** Effect of *Salmonella* on constitutively-AKT and autophagic pathway.** The ASH-3 (**A**) and KMH-2 (**B**) cells (10^6^ cells/well) were placed into 6-well plates and incubated at 37 °C for 24 hours. The cells were transfected with control or constitutively active AKT plasmids (5ug) at 37 °C for 6 hours and then infected with 0 or 100 MOI *Salmonella* for 90 min. The expression level of AKT/mTOR/p70S6K proteins and autophagic marker in cells were analyzed by Western blotting. β-actin was used as an internal loading control. Quantification histograms are presented beneath each Western blotting plot. Data are expressed as the mean ± SD of hexaplicate determinations. Each experiment was repeated three times with similar results.

To confirm the role of AKT pathway in *Salmonella*-induced autophagy, a constitutively active form of AKT plasmids were transfected into ASH-3 (Figure 4A) and KMH-2 (Figure 4B), and the expression of LC3 and AKT/mTOR/p70S6K signaling pathway were monitored by Western blotting. The overexpression of active AKT reduced the conversion of LC3 and increased the expression of AKT/mTOR/p70S6K phosphorylation, demonstrated that the AKT signaling pathway is the upstream pathway involved in *Salmonella*-induced autophagy. These results can also be clearly observed in the quantification histogram below. Our results point out that *Salmonella* may play a role as an autophagy inducer via suppression of AKT/mTOR/p70S6K signaling pathway in ATC.

### *Salmonella* targeted tumor, induced autophagy and increased infiltrating immune cell* in vivo*

**Figure 5:**
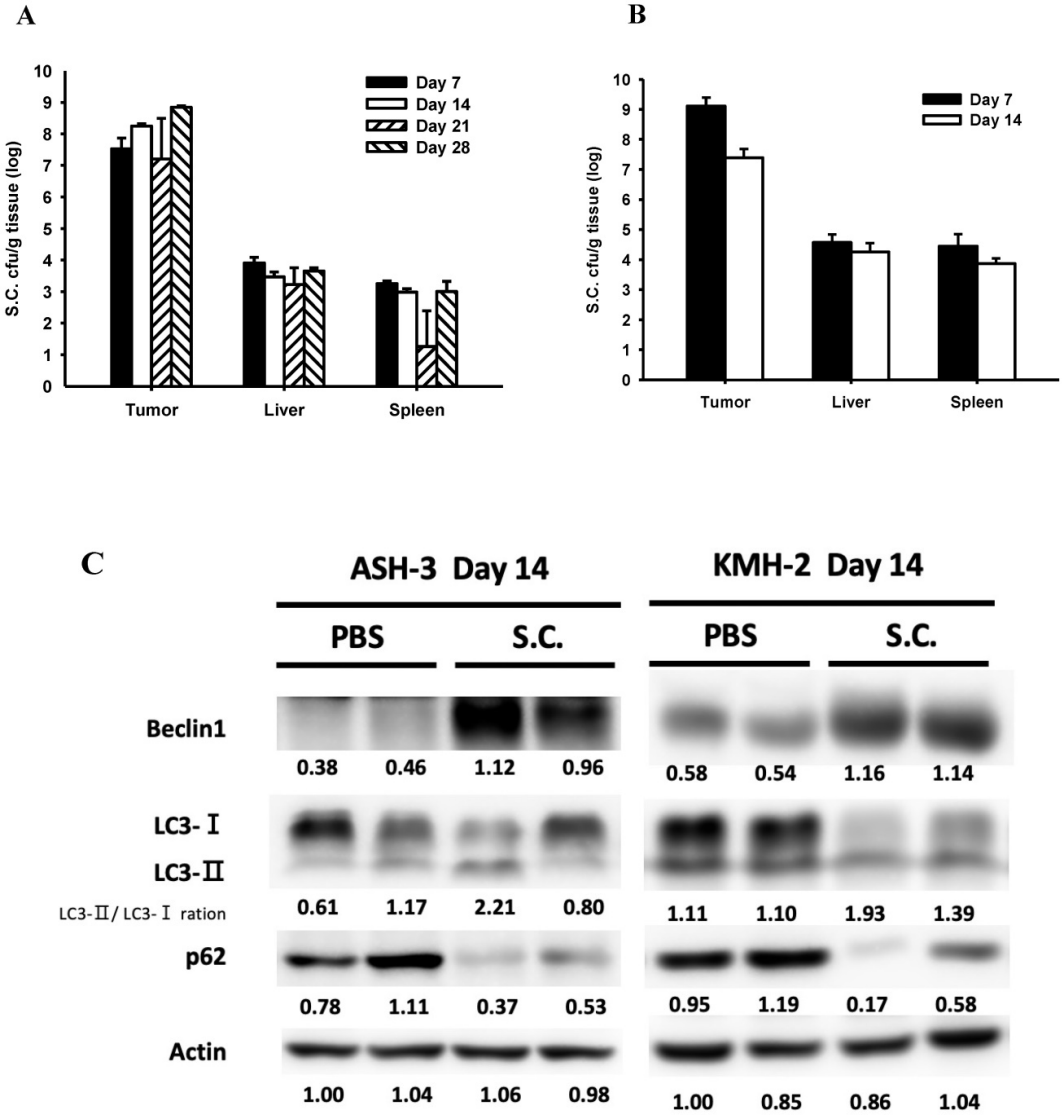

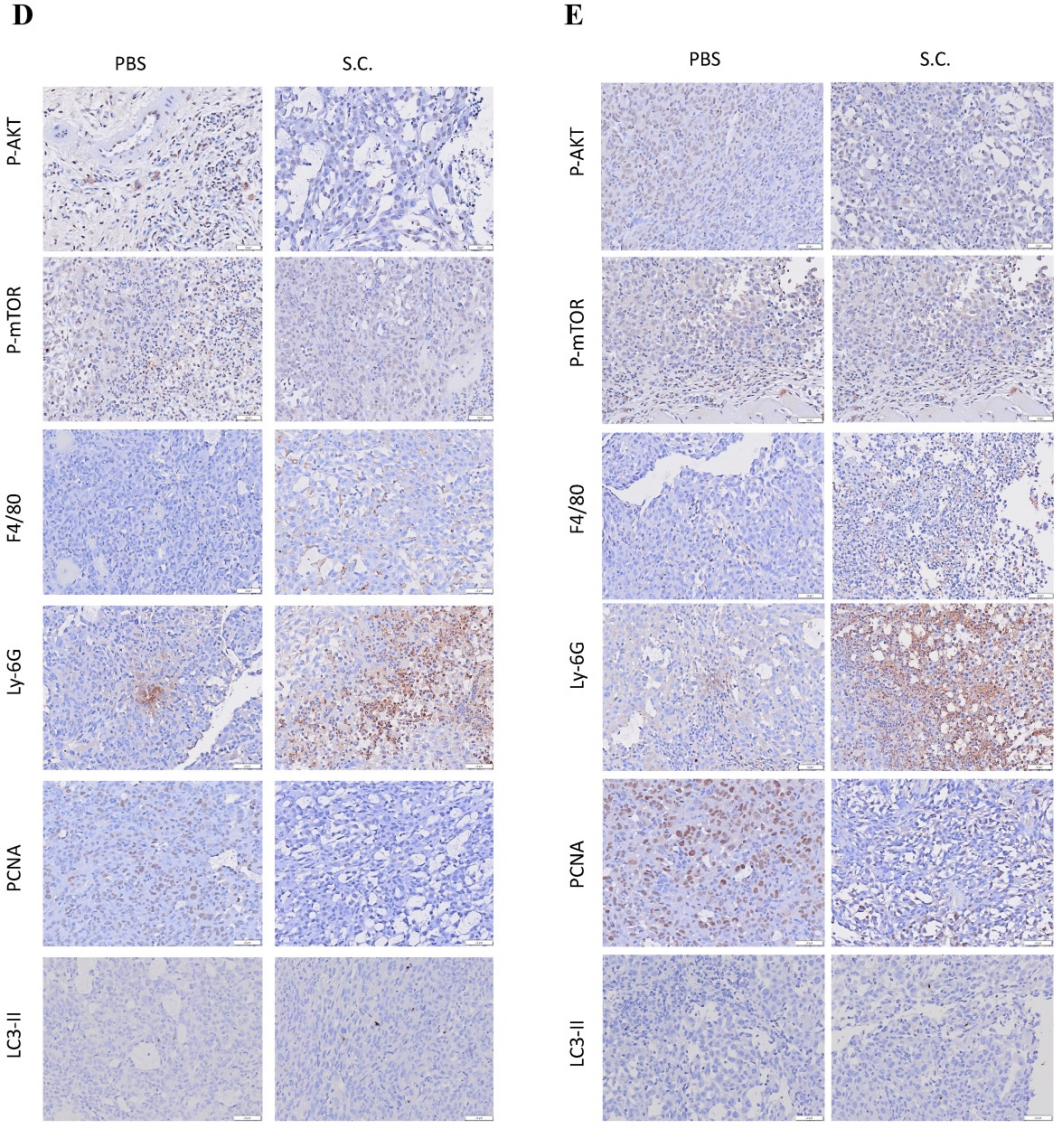
***Salmonella* (S.C.) targeting tumor potential and induction of tumor autophagy *in vivo*.** (**A**) The mice bearing ASH-3 were injected intraperitoneally with *Salmonella* (2×10^6^ cfu) at day 0. The mice were sacrificed at day 7, 14, 21, and 28 to calculate the amounts of *Salmonella* in the tumor, liver and spleen. (**B**) The mice bearing KMH-2 were injected intraperitoneally with *Salmonella* (2×10^6^ cfu) at day 0. The mice were sacrificed at day 7 and 14 to calculate the amounts of *Salmonella* in the tumor, liver and spleen. Each value represents mean ± SD from 3 mice. (**C**) Tumors were excised on day 14 and the protein expression of autophagic markers in tumor cells derived from *Salmonella*-treated mice or control mice was determined by Western blotting. Inserted values indicated relative protein expressions in comparison with β-actin. Tumor were excised at day 7 (**D**) and day 14 (**E**), the expression of P-AKT, P-mTOR, F4/80, Ly-6G, PCNA, and LC3-II were detected by immunohistochemistry. Scale bar = 30 µm (200X).

To study the effects of *Salmonella* treatment in tumor-bearing mice, we first detected *Salmonella* distribution after intraperitoneal injection with 2 × 10^6^ colony-forming units (cfu) of *Salmonella* in mice bearing ASH-3 (Figure 5A) or KMH-2 (Figure 5B) tumors. Here, observed that the amount of *Salmonella* in tumors is dramatically higher at about 1 million times in the tumor than in blood, liver, and spleen at all-time points. In fact, the amount of *Salmonella* in blood was undetectable on day 7 in ASH-3 bearing mice. On day 7 in KMH-2 bearing mice, there were an average of 780 cfu/ml *Salmonella* in blood, and on day 14 no bacteria were detected in blood. Then, we collected the tumors from mice at different time points and analyzed tumor tissues by Western blotting. In Figure 5C, the protein abundance from tumor lysates on day 14 showed that *Salmonella* increased the conversion of LC3-I to LC3-II, enhanced the expression of beclin-1 and decreased the expression of p62 on both ASH-3 and KMH-2. Moreover, we observed the mice-bearing ASH-3 tumor which were sacrificed on day 7, 21, and 28, tumor samples also showed the identical result that decreased the expression of p62 and increased the conversion of LC3-I to LC3-II and the expression of beclin-1 (Figure S3A, B, and C). In mice-bearing KMH-2 tumor showed the similar results in LC3 and p62 on day 7 (Figure S3D). Therefore, the findings indicated that *Salmonella* can preferentially colonize the tumor to induce autophagy *in vivo*. Then we analyzed the cell proliferation marker PCNA, autophagy marker LC3-Ⅱ, the expression of AKT/mTOR, and infiltrates of macrophages (F4/80) and nutrophils (Ly-6G) in tumors form ASH-3 bering mice treated with *Salmonella* or PBS were analyzed on day 7 and day 14 after infection by immunohistochemistry. Figure 5D and E show the results of immunohistochemical staining. For the expression of P-AKT and P-mTOR, tumors form *Salmonella*-treated mice appeared less expression than PBS-treated counterparts both on day 7 and day 14. As for immune cell infiltrating in the control tumors, abundant macrophages and neutrophils were detected, whereas the tumors on day 14 is more significant than on day 7. Furthermore, tumors from *Salmonella*-treated mice exhibited much lower proliferation than PBS-treated counterparts, while on day 14 was more significant than on day 7. As for autophagy maker, LC3-Ⅱ expression was decreased in the tumors of *Salmonella*-treated group compared with PBS groups in both day 7 and 14. As shown in Figure 5D and E, the expression of P-AKT, P-mTOR, and LC3-Ⅱ has changed slightly in immunohistochemical staining but in the Western blotting results showed more evident in contrast. Furthermore, the significant differences in macrophages, neutrophils, and proliferation expression may give reasonable suggestions for future research directions or treatment strategies. Taken together, these results reveal that *Salmonella* was capable of inhibiting tumor prolifiration and enhancing immune cells infiltration within tumors.

### *Salmonella* inhibited tumor growth* in vivo*

**Figure 6:**
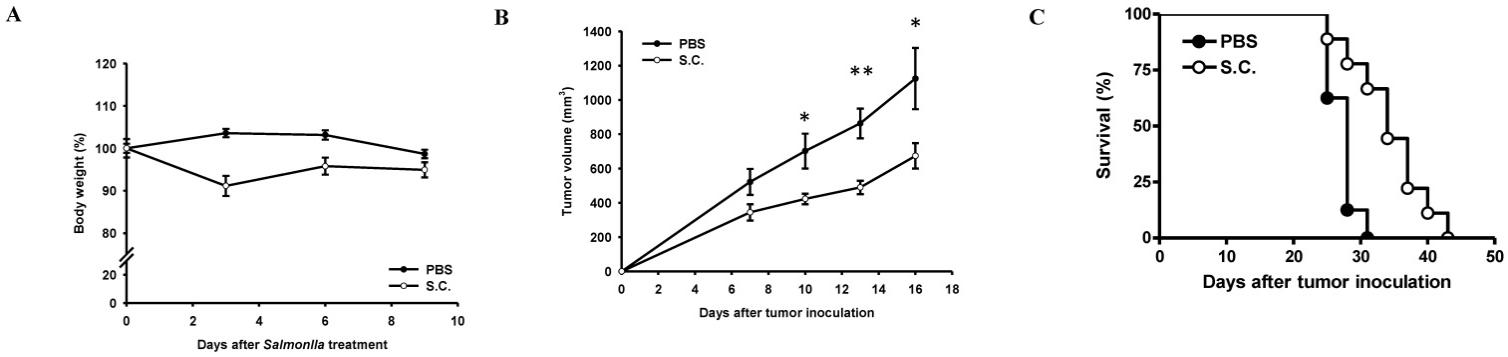
** Antitumor effects of *Salmonella* on mice bearing ATC.** BALB/c nude mice were inoculated subcutaneously ASH-3 (10^7^) at day 0 and intraperitoneal injected *Salmonella* (2×10^6^ cfu) or PBS at day 7. (**A**) The body weight (mean ± SD, n = 10) were compared with PBS group. (**B**) Tumor volumes (mean ± SD, n = 10) in mice bearing ASH-3 tumors were compared with PBS group. *P < 0.05; **P < 0.01. (**C**) Kaplan-Meier survival curves of ASH-3 bearing mice with *Salmonella* or PBS are shown (*p* < 0.01 for mice bearing ASH-3 treated with *Salmonella versus* PBS).

To validate tumor growth inhibition *in vivo*, BALB/c nude mice were inoculated subcutaneously ASH-3 (10^7^) at day 0 and intraperitoneal injected *Salmonella* (2×10^6^ cfu) or PBS at day 7. To analyze the safety of the *Salmonella* therapy, the body weight of mice was measured every 3 days. In Figure 6A, the weight of tumor-bearing mice was reduced by 8.87% on day 3 after treatment with *Salmonella*, but gradually recovered to normal weight on day 9. The antitumor effects of Salmonella were assessed in terms of tumor growth and survival of mice bearing ASH-3 tumor cells. In Figure 6B, the tumor volume from the ASH-3 tumor model was significantly inhibited after *Salmonella* treatment compared with the PBS group on day 10. In addition, the delayed tumor growth of ASH-3-bearing mice treated with *Salmonella* was also significantly observed. Figure 6C showed the survival of ASH-3-bearing mice treated with *Salmonella* were extended in comparison with the PBS group. Overall, our findings suggest that *Salmonella* can inhibit tumor growth and enhance the survival of ASH-3 tumor-bearing mice. Further, we attempted to use KMH-2 cells in mice; unfortunately, we were unable to collect enough tumor-bearing mice to measure the efficiency of *Salmonella* in this system even in the presence of Matrigel upon tumor inoculation.

## Discussion

The primary option of traditional treatment for thyroid tumors is surgery and always supplemented by RAI therapy combined with TSH suppression. Thyroid cells have the ability to absorb iodine compared with other cells; taking advantage of this cellular process by employing RAI treatment will promote cell destruction of the cells that has absorbed the radioactive material, i.e., iodine 40. However, in poorly differentiated thyroid cancer (PDTC), the prognosis is poor because of the lack of differentiation and the high propensity to metastasize 2. Among thyroid cancers, ATC is the most aggressive and has a poor survival rate and prognosis 41. Local recurrence occurs in up to 20% of treated patients with thyroid cancer, and the recurrence state may manifest as dedifferentiation and reduced iodine uptake, rendering adjuvant radioactive iodine ineffective. At present, the molecular mechanism for the treatment of iodine-resistant thyroid cancer is being rapidly developed. For example, studies on ATC have shown that the BRAF/MEK inhibitor has a significant effect on BRAF mutant ATC, or there are also targeted inhibitors for MAPK or PI3K/mTOR/AKT that are found to have a favorable response 42. However, these methods still have adverse effects, or the survival rate is not significantly improved. Moreover, the major limitation in discovering effective treatments for ATC is the rarity of the disease. Due to the rarity of ATC and associated with the shorter survival time of ATC patients, we use nude mice for *in vivo* experiments and corresponding results were obtained in this study. These experimental results may provide a basis or bridge for future clinical research in the treatment of thyroid cancer patients.

Over the past few decades, *Salmonella* has been extensively studied for the treatment of various tumors 43. The use of *Salmonella* has many advantages over traditional therapies, such as the potential of bacteria to prevent tumor metastasis and the ability to resensitize chemically resistant tumor cells aside from its innate oncolytic properties. Furthermore, the characteristics of this facultative anaerobic bacteria that enables it to aggregate and preferentially grow in hypoxic tumor environment, makes it a best fit for targeted tumor therapy 12. Indeed, our mice experiments showed that the number of *Salmonella* in tumors is about 1 million times higher than in other tissues (Figure 5). Nonetheless, the potential side effects of *Salmonella*-mediated therapy limits the use of this bacteria as a treatment, most notably is the host immune response that targets *Salmonella* itself. This problem, however, has also been resolved using attenuated and modified *Salmonella* as in previous studies 44. When *Salmonella* enters the tumor microenvironment, it competes for nutrients and triggers immune cell infiltration into tumor tissue and initiates tumor cell self-destruction, while delaying tumor cell migration 45. In this study, it was found that *Salmonella* can activate the autophagy signaling pathway in ATC by down-regulating AKT/mTOR/p70S6K. In addition, *Salmonella* inhibits tumor cell growth by many mechanisms, such as induction of cell death through apoptosis 26. The nude mice we used in this experiment lacks thymus gland, therefore, cannot produce immune cells such as T cells. Our recent study found that *Salmonella* inhibits tumor metastasis by inhibiting AKT/mTOR and downregulating SNAI1 to induce macrophage reprogramming toward an M1-like phenotype in tumors 13. More previous studies have also found that *Salmonella* eliminates tumors by recruiting neutrophils to infiltrate colonized tumors 45. Other studies have mentioned that ATC usually originates from and can coexist with differentiated thyroid cancer, but may also develop *de novo* 9. If *Salmonella* can effectively treat ATC patients, it is also possible that it will be effective in different types of thyroid cancer. It may also change the treatment strategy for thyroid tumors, avoiding unnecessary treatments such as thyroidectomy, and improve the prognosis.

In our study, the use of *Salmonella* to treat ATC and it was revealed, based on our findings, that the treatment substantially inhibited tumor growth. *In vivo*, we found that large numbers of *Salmonella* preferentially targeted tumor tissue, initiated immune responses, and regulated anti-tumor immune mechanisms. Although there is a low probability for KMH-2 cells to successfully grow in nude athymic mice, resulting in some of our experiments unable to proceed using our system, other groups have successfully used NOD/SCID/γcnull (NOG) mice to grow KMH2 tumors by subcutaneous injection, so this portion of the study needs further investigation 46. This study not only explored the effect of *Salmonella* on tumors, but also dissected a mechanism that adds to the understanding of ATC, a rare thyroid cancer, and provided more promising treatment methods and strategies.

## Supplementary Material

Supplementary figures.
