# Supplementary material for: Downregulation of AKT/mTOR signaling pathway for Salmonella-mediated autophagy in human anaplastic thyroid cancer
Source: J Cancer. 2022 Sep 6;13(11):3268–79. doi: 10.7150/jca.75163 (PMC9475365; doi:10.7150/jca.75163)
Supplement: Supplementary file 1 — Supplementary figures. [file jcav13p3268s1.pdf]

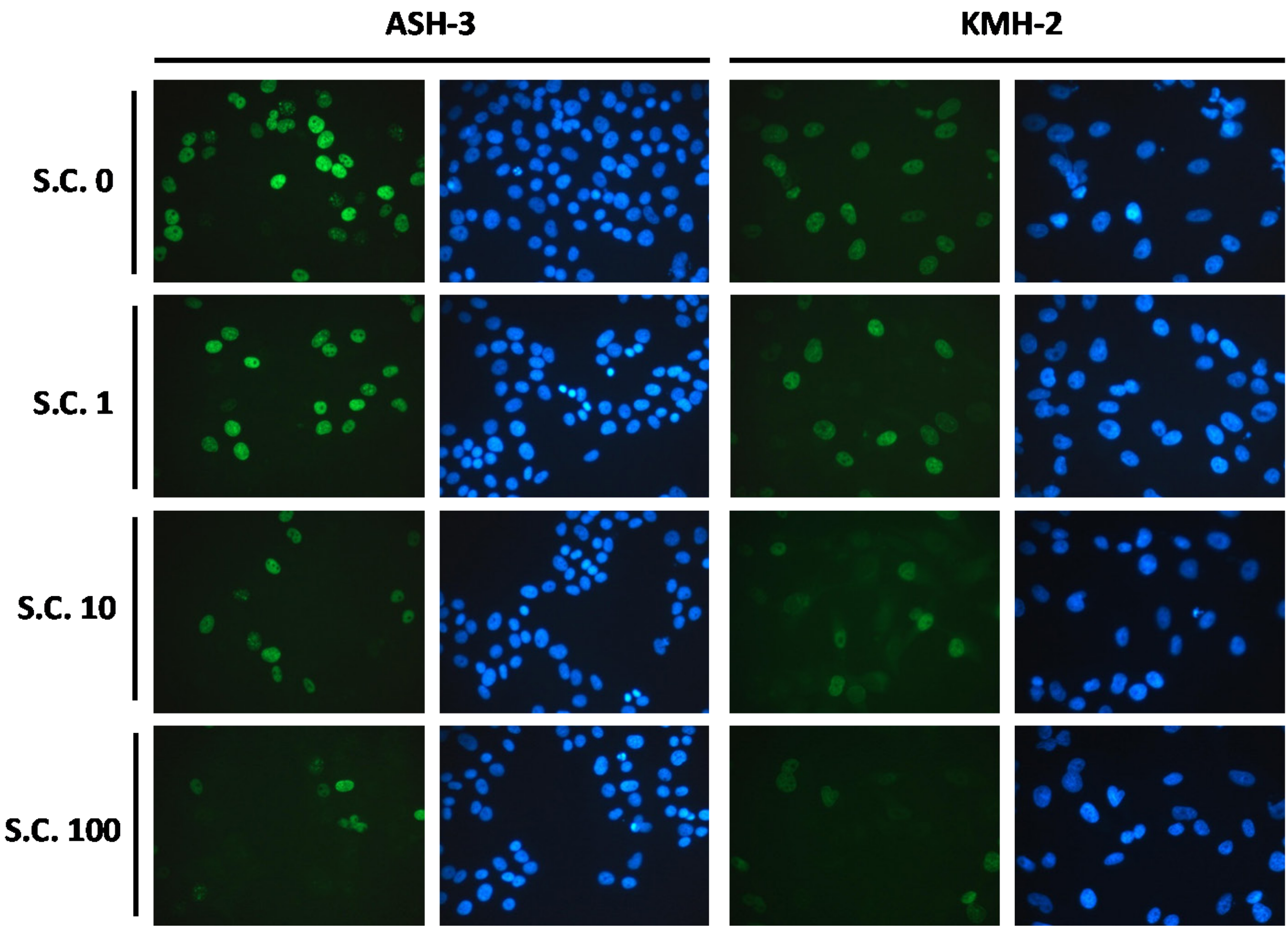


**Figure S1. Detection of BrdU-labeled in ATC cells treated by *Salmonella*.** The ASH-3 and KMH-2 cells ($5 \times 10^5$ cells/well) were placed into 6-well plates and incubated at 37°C for 24 hours and then the cells were infected with 0-100 MOI of *Salmonella* for 90 min. Then the cells were fixed and stained for BrdU (green) and nuclei were counterstained with Hoechst 33342 (blue). The cells were counted under a fluorescence microscope.

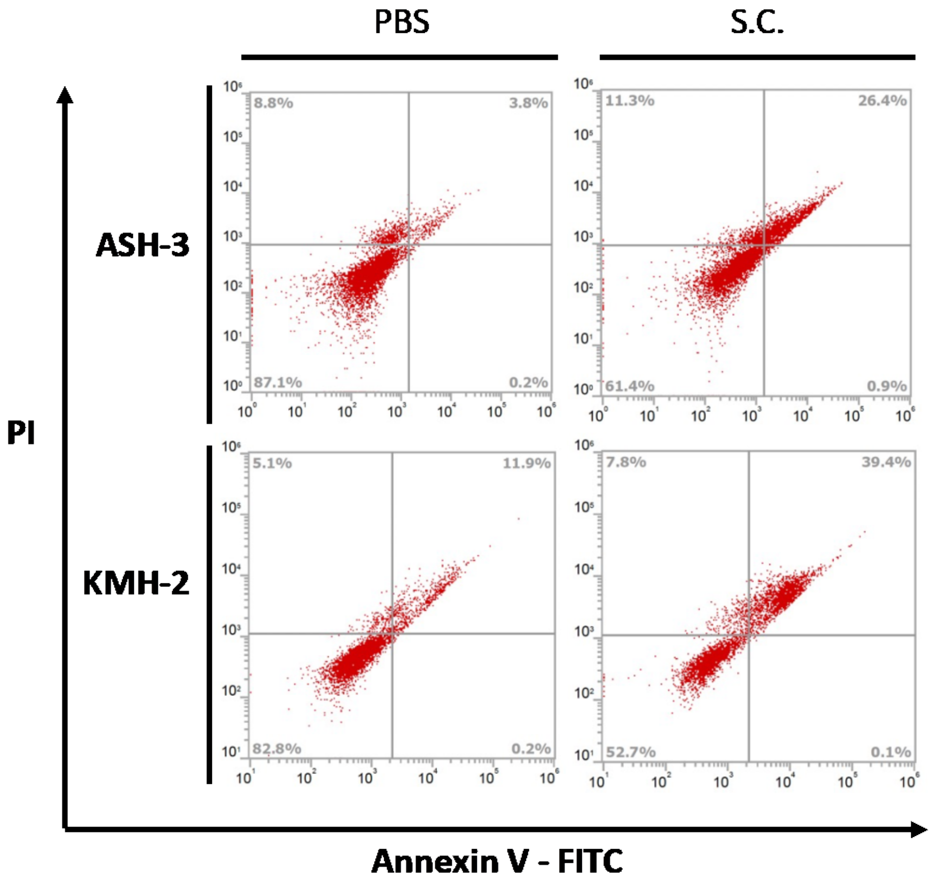


**Figure S2. *Salmonella* induces cell death in ATC cells.** The ASH-3 and KMH-2 cells ($5 \times 10^5$ cells/well) were placed into 6-well plates and incubated at 37°C for 24 hours and then the cells were infected with 100 MOI of *Salmonella* for 90 min. The death cells were examined with flow-cytometry.

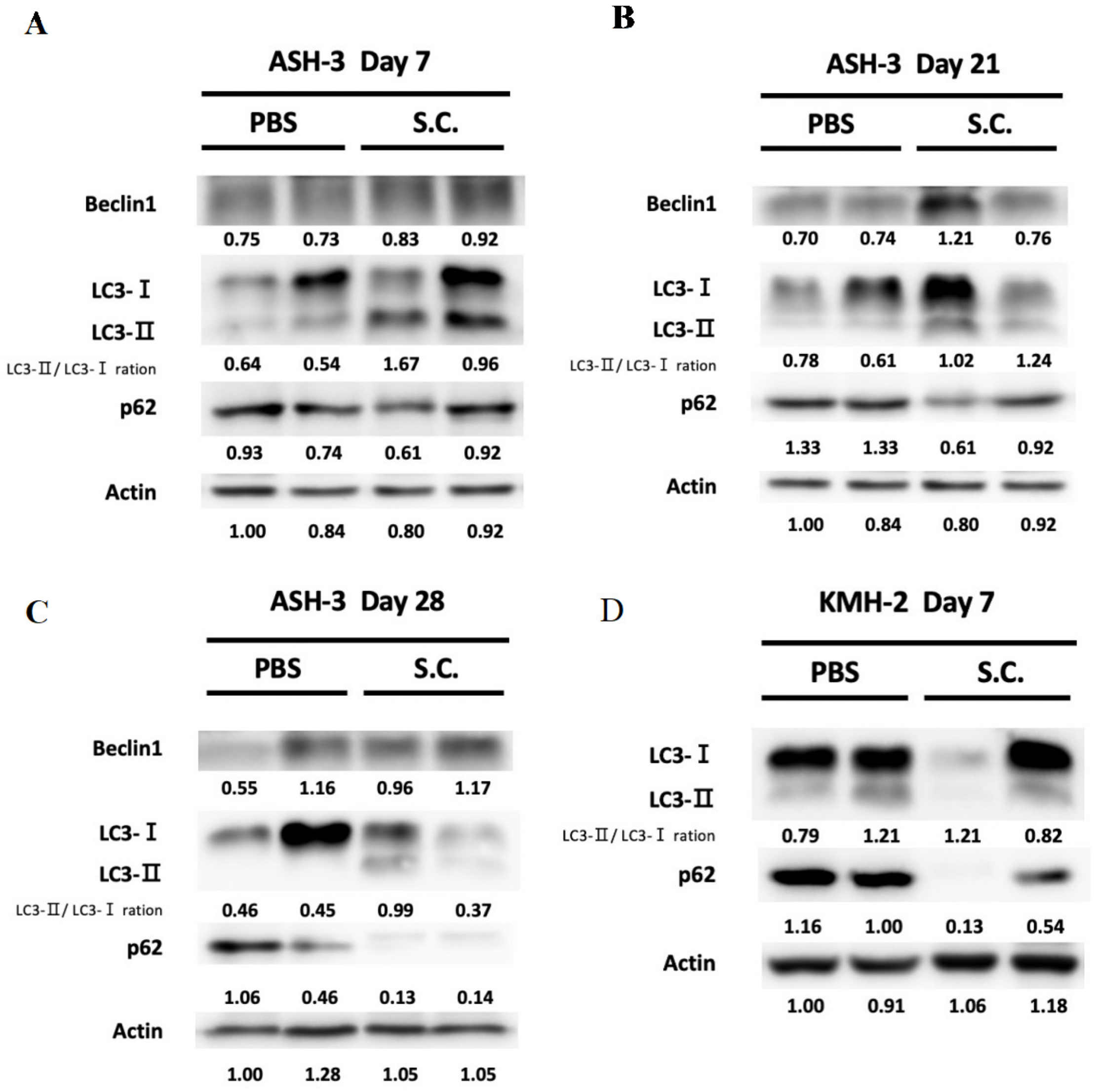


**Figure S3. *Salmonella* (S.C.) induction of tumor autophagy *in vivo*. (A-C)** The mice bearing ASH-3 were injected intraperitoneally with *Salmonella* ($2\times10^6$ cfu) at day 0 and the mice were sacrificed at day 7, 21, and 28. **(D)**The mice bearing KMH-2 were injected intraperitoneally with *Salmonella* ($2\times10^6$ cfu) at day 0 and the mice were sacrificed at day 7. Then tumors were be lysed to analyze the protein expression of autophagic markers in tumor cells derived from *Salmonella*-treated mice or control mice was determined by Western blotting. Inserted values indicated relative protein expressions in comparison with **β**-actin.
